# Mosquitoes Trade Fertility for Immune Defense against Malaria

**DOI:** 10.1371/journal.pbio.1002256

**Published:** 2015-09-22

**Authors:** Caitlin Sedwick

**Affiliations:** Freelance Science Writer, San Diego, California, United States of America

## Abstract

A study of *Anopheles gambiae* mosquitoes shows that a molecule involved in defense against the malaria parasite also plays a role in male fertility, identifying a potential evolutionary trade-off between immunity and reproductive fitness. Read the Research Article.

A quick, sharp burst of pain brings a hand clapping down upon an arm or other body part, but even if delivered with alacrity, the blow comes too late. The mosquito has already pierced the skin, injected an anticoagulant, and perhaps taken a sip of blood. In Africa, where the several mosquito species comprising the *Anopheles gambiae* complex thrive, she may also have delivered a gate-crasher past the defensive threshold of her target’s skin: the parasite *Plasmodium falciparum*, the causative agent of malaria. Thus is the mosquito reviled, not only for the legacy of painful welts she leaves behind, but also for her role in spreading disease.

However, the fact is that the *Plasmodium* parasite is just as much an unwelcome guest for mosquitoes as it is for humans, and accordingly, it is subject to attack by the insect’s immune system. Key to mosquitoes’ ability to fend off malarial infection is a glycoprotein called thioester containing protein-1 (TEP1). TEP1, a homolog of the mammalian complement factor C3, is present in the hemolymph (the mosquito’s functional equivalent for blood), where it recognizes and binds to various pathogens—including the *Plasmodium* ookinetes that live in the mosquito midgut. Similar to its mammalian counterpart, C3, TEP1 binding to a pathogen’s surface flags the invader for phagocytosis. It also initiates the activation of a “complement-like cascade” that drives the microbe’s lysis. This simple but effective defense mechanism can render mosquitoes remarkably resistant to *Plasmodium*; yet, despite this, the parasite persists, causing hundreds of thousands of human deaths each year. Now, in their paper published this month in *PLOS Biology*, Julien Pompon and Elena Levashina identify a new role for TEP1 that may help explain why *Plasmodium* remains such a scourge of both mosquito and man.

Because only female mosquitoes take blood meals and transmit the parasite to human populations, until now most studies on TEP1 have focused on how the protein operates during the female’s immune response. However, while using immunofluorescence to survey tissues of male mosquitoes for the presence of TEP1, Pompon and Levashina were surprised to discover that TEP1 is present on both spermatagonia and spermatozoa. This raised the question of why TEP1 might associate with these cells.

Spermatozoa, the end products of spermatogenesis, arise from spermatogonia after multiple rounds of mitotic and meiotic cell division in the testes (Fig 1). The authors observed that the number of TEP1-positive spermatogonia was low in virgin males but increased during the wave of new spermatogenesis that follows immediately after mating. In contrast, the number of TEP1-positive spermatozoa did not increase, indicating that TEP1 is likely important for spermatogonial development.

**Fig 1:**
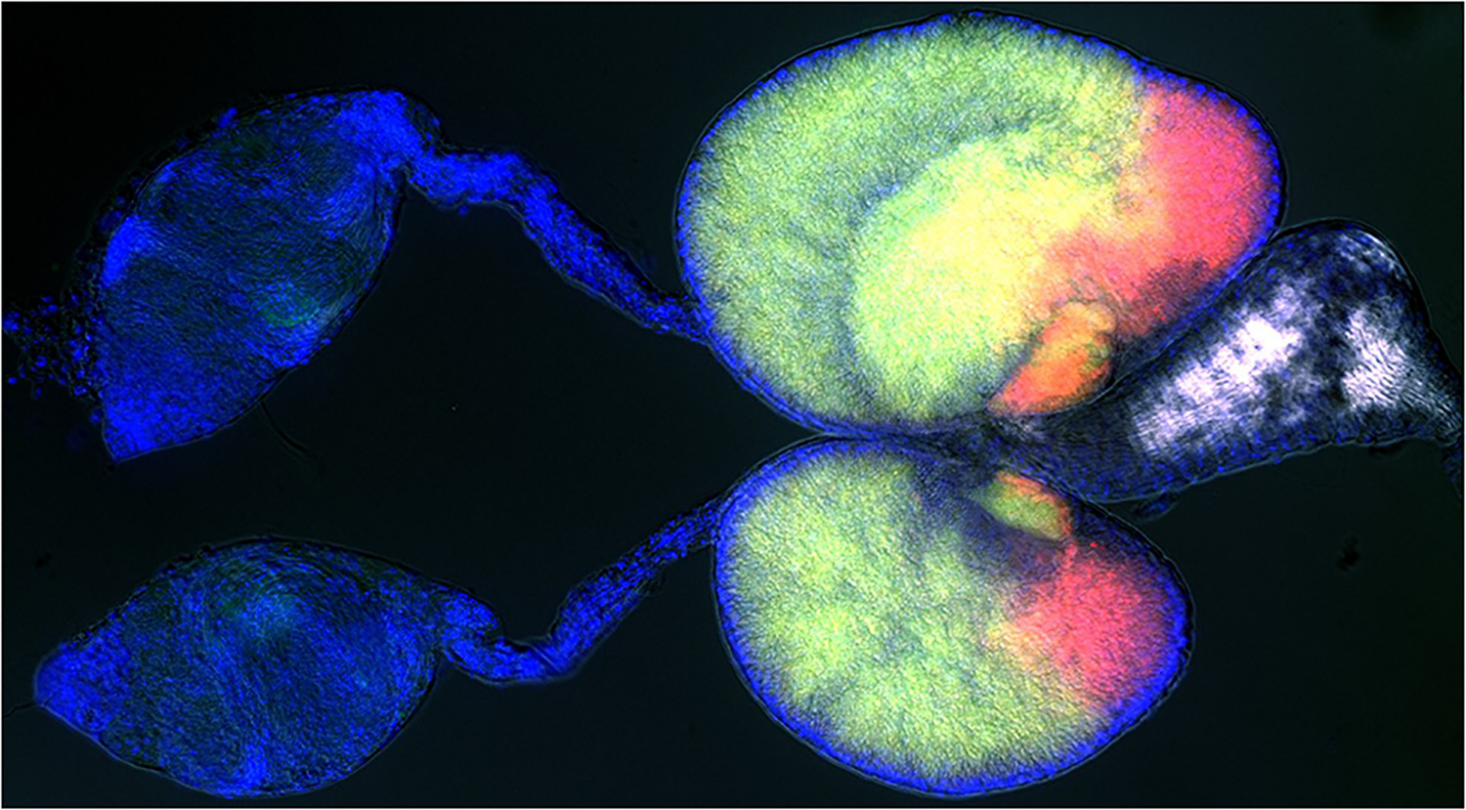
Male reproductive organs of the mosquito *A*. *gambiae*. Sperm development takes place in the testes (shown in blue). Mature sperm travels to the ejaculatory organ (white), where it is mixed with accessory proteins produced by the male accessory glands (characterized by strong green and red autofluorescence). *Image credit: Elena Levashina*.

Spermatogonia tend to accumulate genetic errors during the rapid rounds of cell division that accompany spermatogenesis, necessitating the removal of defective cells by apoptosis. In mammals, apoptotic cells are also removed by complement, but the mammalian testis is a complement-free organ, likely to prevent complement activation on the germ cells induced by autoreactive antibodies. As mosquitoes have no antibodies, their complement-like system could be used for clearance of defective cells in the testes. Because TEP1 is known to serve complement-like functions in mosquito immunity, Pompon and Levashina theorized that it may also be recruited to defective spermatogonia. Indeed, they found that the number of TEP1-positive spermatogonia dramatically increased when mosquito testes were irradiated to induce DNA damage. However, TEP1 binding to defective sperm cells was abolished when the researchers knocked down LRIM1 and HPX2, two components of the complement-like cascade that are known to regulate TEP1 binding to pathogens. Furthermore, knockdown of TEP1 interfered with the removal of DNA-damaged spermatogonia. Together, these data suggest that TEP1 and the complement-like cascade are important for sperm quality control in mosquitoes, likely because TEP1 binding to defective spermatagonia flags them for removal. This conclusion was further reinforced by the authors’ observation that TEP1 is important for male mosquito fertility; mating resulted in fewer offspring after TEP1 was knocked down in males.

Interestingly, prior studies have identified four different alleles of the *TEP1* gene, called *R1*, *R2*, *S1*, and *S2*. Pompon and Levashina wondered if the different alleles might have different impacts on mosquito fertility. In fact, they found that male mosquitoes homozygous for the *S2* allele were more fertile than those homozygous for the other alleles. This is significant because the different *TEP1* alleles are also known to govern mosquito susceptibility to parasite infection: mosquitoes expressing the *R1 TEP1* allele are highly resistant to parasite infection, whereas those homozygous for the other versions of the gene are susceptible. Therefore, mosquitoes expressing the *S2* allele face a trade-off between reproductive fitness and immune resistance to parasites. As Pompon and Levashina note, genes involved in regulating reproduction tend to be under heavy selective pressure, so these data could have interesting implications for the susceptibility of mosquito populations to *Plasmodium* infection—and, in turn, for human health.
